# Development of EST-SSR Markers Linked to Flowering Candidate Genes in *Elymus sibiricus* L. Based on RNA Sequencing

**DOI:** 10.3390/plants9101371

**Published:** 2020-10-15

**Authors:** Yuying Zheng, Zongyu Zhang, Yiyang Wan, Jiaoyang Tian, Wengang Xie

**Affiliations:** The State Key Laboratory of Grassland Agro-ecosystems, Key Laboratory of Grassland Livestock Industry Innovation, Ministry of Agriculture and Rural Affairs, College of Pastoral Agriculture Science and Technology, Lanzhou University, Lanzhou 730020, China; zhengyy18@lzu.edu.cn (Y.Z.); zhangzongyu@lzu.edu.cn (Z.Z.); wanyy19@lzu.edu.cn (Y.W.); tianjy18@lzu.edu.cn (J.T.)

**Keywords:** *Elymus sibiricus* L., SSR development, flowering time, genetic diversity

## Abstract

*Elymus sibiricus* L. is an important cold-season grass with excellent cold and drought tolerance, good palatability, and nutrition. Flowering time is a key trait that affects forage and seed yield. Development of EST-SSR (expressed sequence tag simple sequence repeat) markers based on flowering genes contributes to the improvement of flowering traits. In the study, we detected 155 candidate genes related to flowering traits from 10,591 unigenes via transcriptome sequencing in early- and late-flowering genotypes. These candidate genes were mainly involved in the photoperiodic pathway, vernalization pathway, central integrator, and gibberellin pathway. A total of 125 candidate gene-based EST-SSRs were developed. Further, 15 polymorphic EST-SSRs closely associated to 13 candidate genes were used for genetic diversity and population structure analysis among 20 *E. sibiricus* accessions, including two contrasting panels (early-flowering and late-flowering). Among them, primer 28366, designed from heading date 3a (*HD3a*), effectively distinguished early- and late-flowering genotypes using a specifically amplified band of 175 bp. The polymorphic information content (PIC) value ranged from 0.12 to 0.48, with an average of 0.25. The unweighted pair group method analysis (UPGMA) cluster and structure analysis showed that the 20 *E. sibiricus* genotypes with similar flowering times tended to group together. These newly developed EST-SSR markers have the potential to be used for molecular markers assisted selection and germplasm evaluation of flowering traits in *E. sibiricus*.

## 1. Introduction

*Elymus sibiricus* L. is a perennial, predominantly self-pollinating and allotetraploid (2n = 4x = 28) forage grass of the genus *Elymus*. It is widely distributed in high altitude regions of western and northern China, especially on the Qinghai-Tibetan Plateau [1]. *E. sibiricus* is an important cold-season grass with excellent cold and drought tolerance, good palatability, and forage quality [2]. However, research on breeding and improvement for this species is limited. The quantity of *E. sibiricus* varieties cannot meet the growing demands of ecological restoration and animal husbandry in China [3]. In perennial forage grasses, flowering indicates the transition from vegetative growth to reproductive growth, which affects characters of economic importance such as forage quality and seed yield [4]. Therefore, accurate prediction of flowering is very important for breeding early flowering or late flowering varieties to meet different practical needs [5].

To date, molecular mechanisms for flowering in *Arabidopsis thaliana* [6] and many other species have been extensively studied. The main pathways of controlling flowering in *Arabidopsis thaliana* are the vernalization pathway, photoperiodic pathway [7], gibberellin pathway [8], and autonomic pathway [9]. Several key flowering genes such as, *Flowering Locus T* (*FT*), *Suppressor of Overexpression of Constans 1*(*SOC1*), *Leafy* (*LFY*), and *VERNALIZATION 1* (*VRN1*) have been reported [10]. *FT* gene, located at the intersection of different flowering pathways, plays a key role in plant flowering regulation [11]. *SOC1* encoding a MADS-box transcription factor is highly expressed in leaves and floral meristems [12]. *LFY* gene, an important flowering control factor, integrates external environment and endogenous information, regulates the transformation of inflorescence to flowering meristem, and controls the flowering time under the joint action of multiple flowering pathways [13]. *VRN1*, a MADS-box gene, plays an important role in the development of spikelet and spike, and also affects the flowering stage and plant height in wheat [14]. In addition, Fang et al. [15] investigated the characterization and function of the rice *OsFTL* gene. Overexpression of *OsFTL10* led to early flowering of rice by 2 weeks. Liu et al. [16] reported *TaZIM-A1*, which is an atypical *GATa*-like transcription factor that negatively regulated flowering in wheat. Under long-day conditions, overexpression of *Tazima-A1* led to late flowering in wheat. Kang et al. [17] identified *MsFTa*, an *FT* ortholog in alfalfa, and characterized its function in regulating flowering. *MsFTa* plays a promoting role in alfalfa flowering, suggesting that it may participate in the coding of florigen. Huang et al. [18] revealed the genetic network regulating flowering time, and identified four major flowering candidate genes in orchardgrass based on whole genome sequencing. However, studies on the molecular mechanisms controlling flowering have not been reported in *E. sibiricus*.

As a high-throughput sequencing technique, transcriptome sequencing provides almost complete transcript information of biological tissues or cells [19]. Due to the advantages of sequencing depth, RNA-seq can reveal the overall gene expression of an individual at a given moment and in a given organization more comprehensively [20]. RNA-seq is widely used in non-model plants with limited sequence information, because it focuses on the coding region [21]. With the rapid development of molecular markers, EST-SSR (expressed sequence tag simple sequence repeat) markers derived from RNA-seq can be used to analyze genetic diversity, genetic variation, population structure, and functional studies [22]. Development of EST-SSR markers based on interesting candidate genes related to target traits are more valuable for variety improvement. Singh et al. [23] developed 30 SSR markers based on salt tolerance candidate genes in wheat, and evaluated genetic diversity in salt susceptible and salt tolerant wheat genotypes. Babu et al. [24] developed 24 candidate gene-based SSR markers related to lysine and tryptophan metabolic pathways in maize. These candidate gene-based SSRs is useful in genetic improvement of crop plants. Jespersen et al. [25] identified 29 SSRs related to five summer heat and drought tolerance candidate genes. These candidate gene-based SSR markers were useful in molecular marker-assisted selection of stress tolerance in creeping bentgrass. Lai et al. [26] developed EST-SSR derived from flowering genes and found these markers were highly transferable in 17 *Gossypium* species. Zhao et al. [27] reported EST-SSR loci related to candidate genes for seed shattering may have potential application in identifying trait-associated markers in *E. nutans*.

To better understand the mechanism for flowering in *E. sibiricus*, we developed novel EST-SSR markers derived from putative candidate genes for flowering time based on previous transcriptome data in *E. sibiricus*, and used polymorphic EST-SSRs for genetic diversity analysis of *E. sibiricus* genotypes with different flowering time. Early-flowering and late-flowering germplasms can be distinguished by polymorphic EST-SSRs, which could shorten the breeding process in *E. sibiricus*. These newly developed EST-SSR markers could be a useful tool for genetic improvement and molecular markers-assisted selection of flowering traits in *E. sibiricus*.

## 2. Results

### 2.1. Transcriptome Sequencing, Unigenes Annotation, and Differentially Expressed Genes Analysis

To dissect the molecular mechanism and explore the putative genes related to flowering time in *E. sibiricus*, 18 cDNA libraries were constructed from leaves collected from early flowering genotypes and late flowering genotypes at booting stage, heading stage, and flowering stage. Then, these libraries were sequenced. These Illumina data are available in the Sequence Reads Archive (SRA) with accession number SRX9217303-SRX9217319. A total of 400,453,230 clean reads were obtained after filtering out low-quality reads that had a Q30 percentage greater than 91.56%. The average GC content of clean reads was 55% (Table S1). Then, a total of 477,220 transcripts and 184,735 unigenes were identified in all samples. The average length of transcripts and unigenes was 937.56 bp and 750.13 bp, with an N50 length of 1420 bp and 1195 bp, respectively (Table S2).

After comparing BLAST and HMMER to 184,735 unigenes with COG, GO, KEGG, KOG, Pfam, Swiss-Prot, TrEMBL, Nr, and Nt databases, 166,071 (89.90%) unigenes were successfully annotated. The number and proportion of unigenes corresponding to each database are 15,597 (8.44%), 48,508 (26.26%), 12,379 (6.70%), 37,405 (20.25%), 41,919 (22.69%), 42,321 (22.91%), 90,201 (48.83%), 83,698 (45.31%), and 161,713 (87.54%) (Table S3).

**Figure 1:**
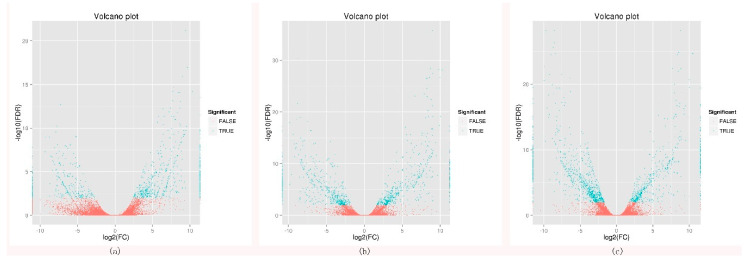
Volcano plot of DEGs (differentially expressed genes) in booting stage, heading stage, and flowering stage of early-flowering and late-flowering accessions. (**a**) Early booting vs. Late booting, (**b**) Early heading vs. Late heading, and (**c**) Early flowering vs. Late flowering.

To investigate the changes in gene expression and explore the putative genes involved in flowering. The differentially expressed genes were identified between the early- and late-flowering genotypes at three development stages. A total of 3526 differentially expressed genes (DEGs) were identified. Among these DEGs, 1715 genes were up-regulated in early-flowering genotypes and 1811 genes were down-regulated in late-flowering genotypes. In the booting stage, 576 genes were up-regulated in early-flowering genotypes and 360 genes down-regulated in late flowering genotypes. In the heading stage, 584 genes up-regulated were up-regulated in early-flowering genotypes and 578 genes down-regulated in late flowering genotypes. In the flowering stage, 555 genes up-regulated were up-regulated in early-flowering genotypes and 873 genes down-regulated in late flowering genotypes. By comprehensive comparison of three DEG sets, the largest number of DEGs was found in the flowering stage (Figure 1).

### 2.2. Frequency and Distribution of EST-SSR Markers

**Figure 2:**
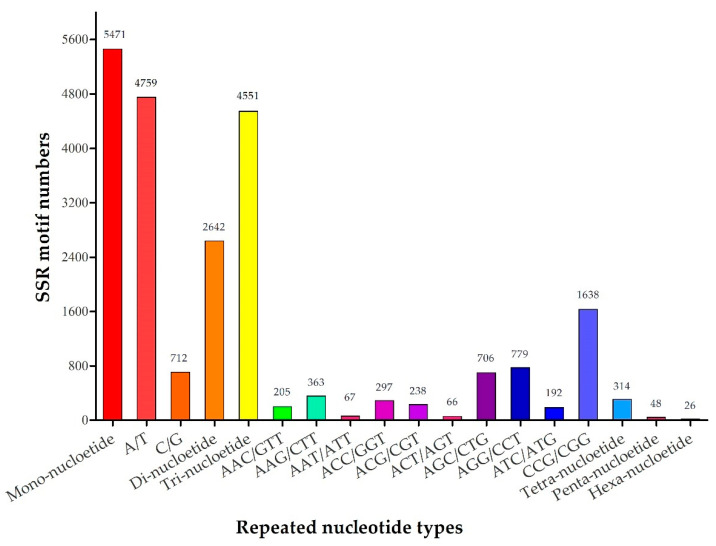
Different types of SSR repeats and their percentage based on RNA-seq.

Among the transcriptome assembly, 13,052 SSRs were identified from 40,639 unigenes and the number of SSR containing sequences was 10,286. The number of sequences containing more than 1 SSR was 2176 and the number of SSRs present in compound formation were 742. The identified SSRs comprised of mono- to hexa-nucleotide repeats with a big difference in quantity (Table 1). Mono-nucleotide repeats were the most abundant (5471, 41.92%), followed by tri-nucleotide (4551, 34.87%), di-nucleotide (2642, 20.24%), tetra-nucleotide (314, 2.41%), penta-nucleotide (48, 0.37%), and hexa-nucleotide repeats (26, 0.20%) (Table 1). Among the SSR motifs, the most dominant mono-nucleotide repeat motif was A/T (4759, 36.46%), followed by tri-nucleotide repeat motifs of CCG/CGG (1638, 12.55%) and AGG/CCT (779, 5.97%) (Figure 2).

### 2.3. Development of Candidate Gene-Based EST-SSR Markers

**Figure 3:**
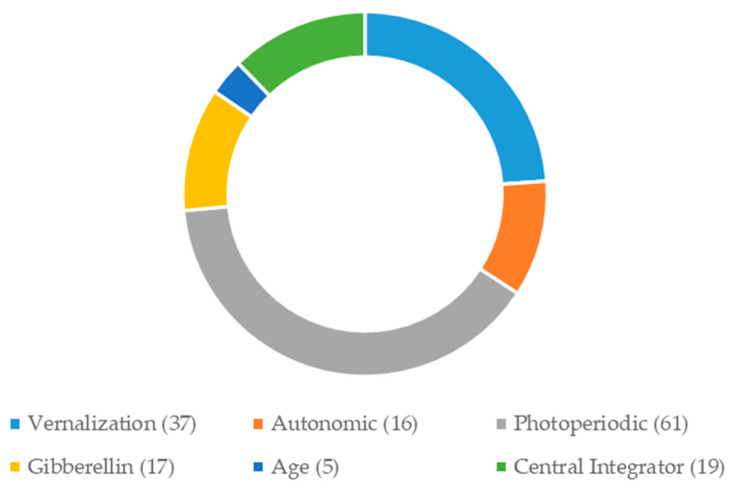
The number of candidate genes involved in six flowering regulatory pathways.

Based on gene function annotation, we selected 155 candidate genes associated with flowering time from 10,591 *E. sibiricus* unigenes (Table S4). These candidate genes involved in vernalization response (6), flowering (31), circadian clock (12), photoperiodic response (4), *CONSTANS-Like* (45), gibberellin response (17), autonomous response (16), age pathways (5), and central integrator (19). Photoperiodic pathways were the most abundant (61, 39.35%), followed by vernalization pathways (37, 23.87%), central integrator (19, 12.26%), gibberellin pathways (17, 10.97%), autonomic pathways (16, 10.32%), and age pathways (5, 3.23%) (Figure 3). A total of 125 pairs of candidate gene-based SSR primers were designed and used to amplify four *E. sibiricus* genotypes with different origins. The unigene sequences used for marker development and primer sequences can be found in GenBank (BankIt2383886: MW 016765–MW 016888, and MW052548).

**Figure 4:**
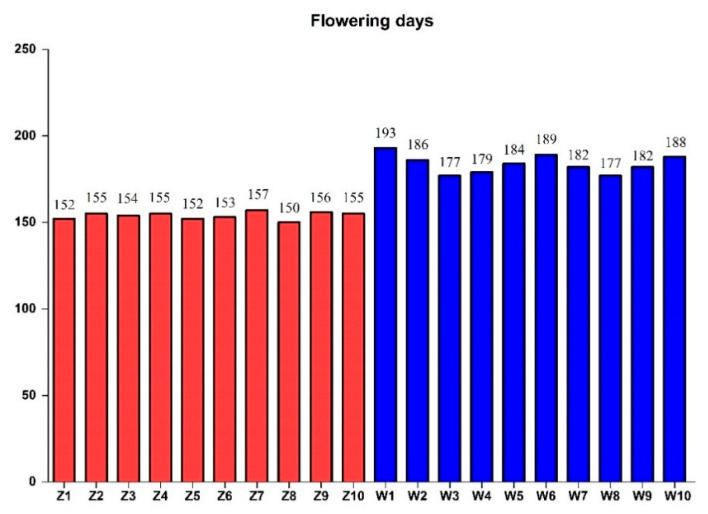
The flowering days of 20 *E. sibiricus* germplasms from 1 January 2018.

Among the 125 designed candidate gene-based EST-SSR (expressed sequence tag simple sequence repeat) primers, 106 pairs of primers generated clear bands with expected size (Table S5). Finally, 15 polymorphic primers were used to analyze genetic diversity among 20 E. sibiricus accessions. Among the 20 E. sibiricus accessions, the flowering time varied from 150 (Z8) to 193 days (W1), with an average of 169 days. The average flowering time for early flowering accessions and late flowering accessions were 154 days and 184 days, respectively (Figure 4).

**Figure 5:**
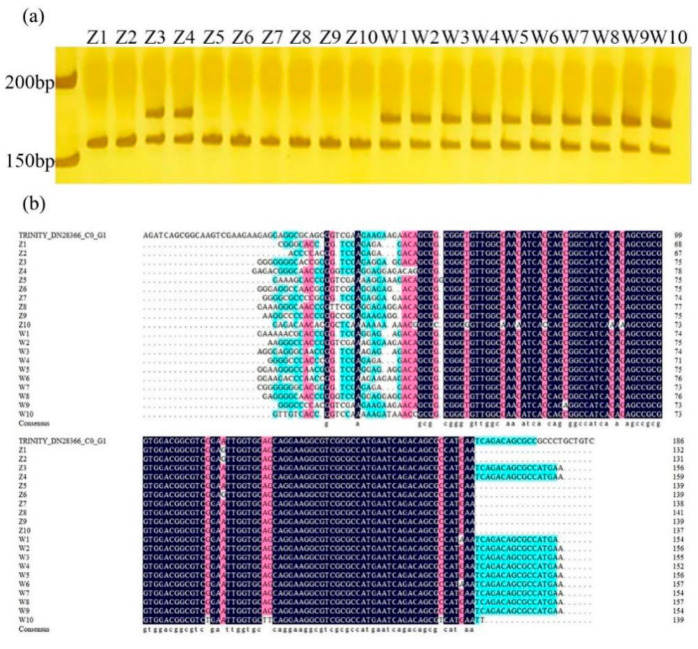
Amplified results of primer 28366 in 20 *E. sibiricus* germplasms. (**a**) The result of PAGE (polyacrylamide gel electrophoresis) of primer 28366 in 20 *E. sibiricus* germplasms. (**b**) Multiple sequence alignment of PCR (polymerase chain reaction) products of primer 28366 in 20 *E. sibiricus* germplasms.

Based on our results, primer 28366 designed from *HD3a* can distinguish early-flowering and late-flowering accessions directly by specifically-amplified bands. The 175 bp band was found in all late-flowering genotypes, but this band was absent in most early-flowering genotypes, except for Z3 and Z4 (Figure 5a). According to the results of PCR (polymerase chain reaction) product sequencing, the Z3 and Z4 genotypes have similar sequence with late-flowering genotypes (Figure 5b) except for some SNP mutations.

### 2.4. Validation of Candidate Gene-Based Specific Primers Authenticity 

**Figure 6:**
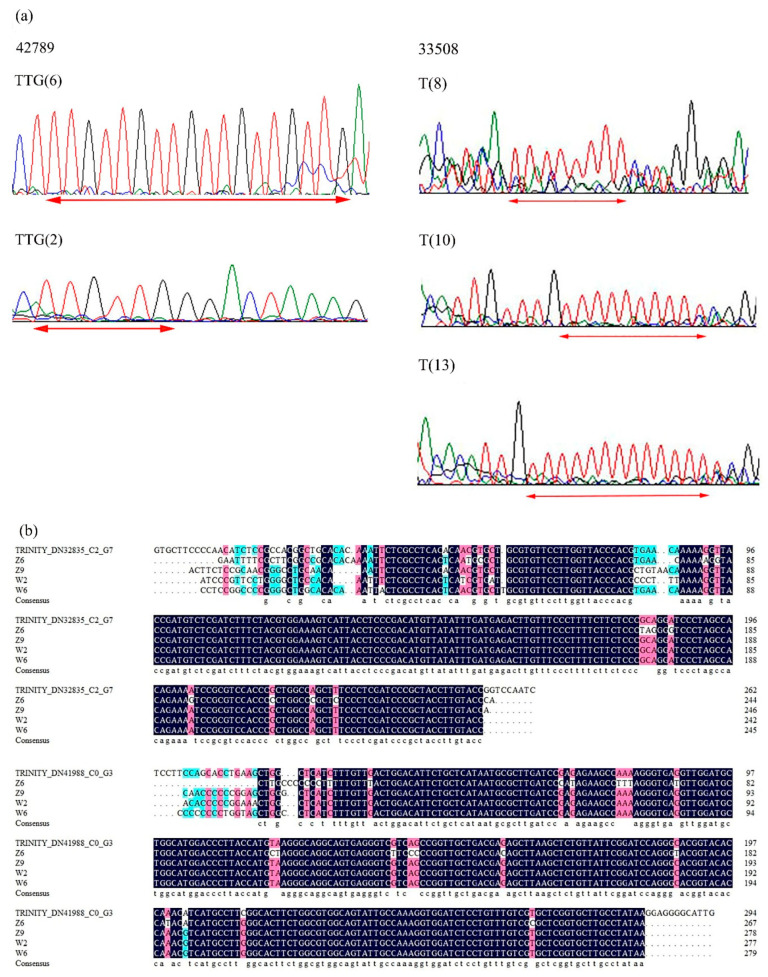
Multiple sequence alignment of RNA-seq and in the late-flowering and early-flowering *E. sibiricus* accessions. (**a**) Comparative electropherogram analysis of two EST-SSR loci (42789 and 33508) in early-flowering and late-flowering *E. sibiricus* accessions. (**b**) Alignment of sequences obtained from selected PCR bands amplified by two primers (32835 and 41988).

To determine the authenticity of candidate gene-based EST-SSR primers, amplicons from 20 *E. sibiricus* germplasms for four EST-SSRs were sequenced. The sequenced alleles from 20 *E. sibiricus* germplasms were homologous to the original SSR motifs from which the primer was designed. According to the sequencing results of expected bands generated from primer 42789, six TTG repeats and two TTG repeats were found in different flowering time genotypes. For primer 33508, *E. sibiricus* accessions with different flowering time had 8 T repeats, 10 repeats, and 13 T repeats, respectively. Marker polymorphisms among the 20 early-flowering and late-flowering *E. sibiricus* accessions were found due to variation in number of repeats of SSR motifs. Alignment of sequences obtained from selected PCR bands amplified by two primers (32835 and 41988) revealed that the base mutation occurs in different *E. sibiricus* accessions. The homology of coding sequences was further verified by multiple sequence alignment (Figure 6).

### 2.5. Genetic Diversity Analysis Using Candidate Gene-Based EST-SSR Markers

All 15 polymorphic primers generated 60 alleles, ranging from 1 to 9 per SSR locus, with an average of 4 alleles. The highest number of alleles was observed in primer 34500. Polymorphic information content (PIC) values of the 15 primers ranged from 0.12 to 0.48, with an average of 0.25. The highest PIC was observed in primer 46865 (0.4767), the lowest PIC was observed in primer 33680 (0.1275). Heterozygosity (Ho) ranged from 0.05 to 1.00 with an average of 0.77. Highest Ho was observed in primer 48198, 34500, 36927, 34261, and 28366 with a mean of 1. The highest gene diversity (He) was in primer 34500 (0.8731), ranging from 0.22 to 0.88 with an average of 0.61 (Table 2).

**Figure 7:**
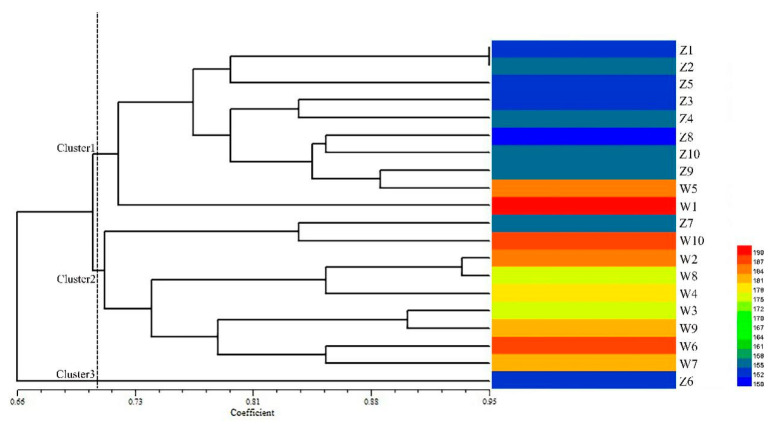
Cluster analysis of 20 *E. sibiricus* accessions with 15 candidate gene-based EST-SSR markers and the heatmap analysis of flowering days of the 20 *E. sibiricus* accessions.

Further, 15 polymorphic EST-SSRs closely associated to 13 candidate genes were used for genetic diversity and population structure analysis among 20 *E. sibiricus* accessions including two contrasting panels (early-flowering and late-flowering). The dendrogram generated through UPGMA (unweighted pair group method analysis) analysis grouped all the 20 *E. sibiricus* accessions into three clusters. Cluster analysis results showed that when the genetic similarity coefficient of 0.71 (Table S6), cluster one contained 8 early-flowering accessions and 2 late-flowering accessions (PI435088 and PI595149). Cluster two contained 8 late-flowering accessions and one early genotype (W630476). Cluster three contained only one early-flowering accession (W610305) (Figure 7). In general, *E. sibiricus* genotypes with similar flowering time tended to group together.

**Figure 8:**
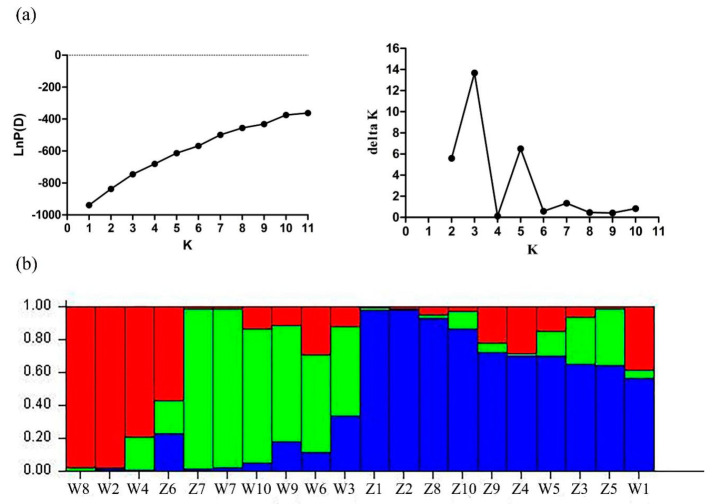
Population structure of 20 *E. sibiricus* accessions inferred from the STRUCTURE program with 15 candidate gene-based EST-SSR markers data set. (**a**) Mean Ln P(D) over 20 runs for each K value, and maximum delta K (ΔK) values were used to determine the uppermost level of structure for K ranging from 1 to 11, where K is three and three clusters; (**b**) three major groups of 20 *E. sibiricus* genotypes. The vertical coordinate of each group indicates the membership coefficients for each genotype. Different codes and corresponding vertical lines represent individual genotype and different colors represent stock.

We employed STRUCTURE-v2.3.2 software (Stanford University, CA, USA) to analyze the population structure among 20 *E. sibiricus* accessions with different values for K (from 1 to 11). Generally, accessions with similar flowering time tended to group together, which was similar to cluster analysis results. When K = 3, the optimal number of groups was three based on maximum likelihood and delta K (ΔK) values. Group 1 in red contained three late-flowering accessions and one early-flowering accession (W610305). Group 2 in green contained five late-flowering accessions and one early-flowering accession (W630476). Group 3 in blue contained eight early-flowering accessions and two late-flowering accessions (PI435088 and PI595149). The flowering candidate gene-based EST-SSR markers may have the potential to distinguish the *E. sibiricus* genotypes with different flowering time (Figure 8).

## 3. Discussion

### 3.1. Candidate Gene-Based EST-SSR Markers with Flowering Time for Marker-Assisted Selection

Flowering time is a turning point from vegetative growth to reproductive growth, which is one of the important factors determining plant productivity and adaptability to the environment in different latitudes [28]. Early-flowering germplasms have more time and resources for reproductive growth because of earlier flowering, so they have a higher seed yield. Late-flowering germplasms, have higher biomass, which is not conducive to seed production, because they need more time and resources for nutrient growth after flowering [29]. The development of early-flowering or late-flowering varieties depends on the accurate evaluation of flowering time. It is difficult to identify flowering time accurately by phenotypic trait due to environment factors. Compared with phenotypic identification, candidate gene-based DNA markers have great potential to improve the efficiency and accuracy of traditional plant breeding through marker-assisted selection (MAS) [30].

Now, the development of transcriptome sequencing technology provides an opportunity for plant genome analysis. With the potential of high throughput, high precision, and low cost, next-generation sequencing technology (NGS) has been widely used in the qualitative and quantitative analyses of transcriptome and has been successfully applied to many plant species [31]. SSRs are classified into genomic-SSR and EST-SSR based on the original sequences used to identify simple repeats [32]. Compared with genomic SSRs, EST-SSR markers have a higher rate of transferability owing to the regions being more evolutionarily conserved than non-coding sequences [33] and have a higher probability of being functionally associated with differential gene expression [34]. The EST-SSR markers based on candidate genes are more likely to be associated with important agronomically traits. *E. sibiricus* is the non-model herbage of *Elymus* genus that does not have whole genomic sequences. Hence, the development of EST-SSR markers based on candidate genes is more suitable for the study of flowering mechanism and improvement of desired trait in *E. sibiricus*.

Kumar et al. [35] identified 13 EST-SSRs significantly associated with flowering time in a diverse panel of 96 accessions of lentil germplasms, and used them as functional markers in the lentil breeding program to develop short duration cultivars. In addition, candidate gene-based EST-SSR explained large phenotypic variation (2.3–21.8%) compared to genomic SSR markers (2.1–10.2%). Li et al. [36] designed 51 SSR primers based on flowering candidate genes and successfully applied them to amplify in 14 *Paphiopedilum orchids* genotypes. These valuable EST-SSR markers were important and useful for novel gene discovery and marker-assisted studies in *Paphiopedilum*. In this study, 15 EST-SSRs closely associated with flowering candidate genes were used for germplasms identification and genetic diversity. Based on our results, most genotypes with different flowering time can be distinguished by EST-SSR markers, especially primer 28366, which can identify all 10 late-flowering genotypes and 8 early-flowering genotypes. Two genotypes, Z3 and Z4, were identified as early-flowering genotypes by flowering time measurement, but they were identified as late-flowering genotypes by specific amplified bands. This indicates that the reliability of candidate gene-based EST-SSR markers was higher than that of phenotypic observation. Primer 28366 was designed from *HD3a* gene related to the central integrator. The photoperiod sensitive gene *HD3* was initially detected as a heading date-related quantitative trait locus on chromosome 6 of rice. The *HD3* region contains two tightly connected loci, *HD3a* and *HD3b*. *HD3a* is a rice ortholog of the *Arabidopsis FT* gene and the *Kasalath* allele of *HD3a* promoted heading under short-day conditions [37,38]. Armstead et al. [39] reported a genetic co-spectral region between rice and ryegrass, including the *HD3* heading-date QTL and a major QTL of rice, accounted for 70% variation. In addition, newly developed markers related the other flowering candidate genes in this experiment have been reported in other species, such as *ELF3*, *GID1*, *GA2OX6*, *XCT*, and *GIGANTEA* [40,41,42,43,44]. In future studies, we will explore the function and regulatory mechanism of these genes in *E. sibiricus* by studying the effect of nucleotide variation of these genes on flowering by sequencing of candidate gene-based markers.

### 3.2. Genetic Diversity of Candidate Gene-Based EST-SSR Markers

Different genetic parameters are important indicators of the origin, evolution, and distribution of new SSR markers. Moreover, they are also important means to measure SSR markers, indicating the existence of moderate heterozygosity in the tested germplasm [24]. In this study, repeated nucleotide types from mono- to hexa-nucleotide were detected in 13,052 identified SSRs from 40,639 unigenes, representing 32.12% of the total detected unigenes. The significant difference was among motif types and frequencies existing in EST-SSRs [45]. In this study, mono-nucleotide repeats were the most abundant SSR motifs (41.92%), followed by tri-nucleotide (34.87%), which was the same as in *Melilotus* [46]. However, di-nucleotide repeats are the most abundant SSR motifs in tall fescue [34] and *Lolium multiflorum* [47], tri-nucleotide repeats are the most abundant SSR motifs in *E. sibiricus* [48] and *Medicago sativa* [49].

Genetic diversity is a key determinant of plant germplasm allocation and genetic improvement. The polymorphic information content (PIC) ranged from 0.12 to 0.48, with an average of 0.25 for 20 *E. sibiricus* accessions, which was similar to *E. nutans.* The PIC varied from 0.220 to 0.370, with an average of 0.318 [27]. However, the value of PIC was lower than barely (the PIC ranged from 0.08 to 0.75 with a mean of 0.46) [50], common vetch (*Vicia sativa* subsp. *sativa*) (the PIC ranged from 0.09 to 0.98) [51], and *E. sibiricus* (the PIC ranged from 0.39 to 0.81) [48]. The reason for this result may due to that EST-SSR primers designed for flowering candidate genes were relatively conservative. A further genome-wide set of SSR markers may result in a higher polymorphism. The dendrogram results showed that these newly SSR markers had the potential to be used for distinguishing *E. sibiricus* germplasms with different flowering time. In total, 20 early-flowering and late-flowering accessions were not completely distinguished possibly due to eco-geographical factors such as, altitude, longitude, latitude, and climate conditions. Climate change at different altitudes will affect flowering time [52]. Grenier et al. [53] reported flowering time were highly correlated with latitudinal and racial distributions of landraces that can be affected by day-length variation. Stuerz et al. [54] found that the key climatic factors affecting flowering were humidity and temperature. Mean air temperature explained 81% variation in duration to flowering across sites, which was furthermore significantly influenced by relative air humidity in lowland rice. Burgarella et al. [55] reported flowering time influence from climatic conditions mainly by variation at genes located upstream in the flowering pathways, close to the environmental stimuli. Variables related to annual precipitation were better than other factors (e.g., temperature, altitude, latitude, or longitude) to reflect the constraint of the flowering time gene. In the study, Z3 and Z4 accessions were identified as late-flowering accessions by primer 28366 but these accessions were still grouped into early-flowering accessions. The result may be due to that flowering time is a complex quantitative trait, which is affected by polygene effect [56]. The monogenic expression of the *HD3*, Z3, and Z4 accessions were characterized as late-flowering genotypes. The regulation effect of single gene expression in the whole flowering network is a micro effect. Therefore, Z3 and Z4 accessions were grouped into early-flowering accessions under co-expression of 13 flowering candidate genes. At the same time, candidate gene-based EST-SSRs development in this study were not effective in identifying all the materials, which may be due to the number of selected genes or the effectiveness of the primers. Future, we can develop recombinant inbred lines (RILs) by crossing early-flowering accessions with the late-flowering accessions to detect flowering QTLs. The development of diagnostic markers could facilitate us to understand the genetic control of flowering in *E. sibiricus*.

The newly designed candidate gene-based SSR markers are useful in marker-assisted selection and the genetic improvement of the flowering trait in *E. sibiricus*. According to different production requirements, *E. sibiricus* germplasms with different flowering time can be selected accurately to meet different agricultural needs.

## 4. Materials and Methods

### 4.1. Plant Materials

In total, 20 *E. sibiricus* accessions including 10 early-flowering accessions and 10 late-flowering accessions were selected for candidate genes-based EST-SSR markers development and diversity analysis in this study (Table 3). All plant materials were planted in the experimental field at Lanzhou University, Yuzhong, Gansu, China (latitude 35°34′ N, longitude 103°34′ E, elevation 1720 m). The flowering time of each accession was determined as the number of days from 1 January to the date when the first inflorescence fully emerged from the flag leaf according to the methods described by Xie et al. [57]. Totally, the flowering time of 20 *E. sibiricus* accessions were measured. According to flowering time results, early-flowering accessions were recoded as Z1 through Z10 and late-flowering accessions were recoded as W1 through W10.

### 4.2. Development of Candidate Gene-Based EST-SSR Markers Based on RNA-Seq in E. sibiricus

In our previous study, six *E. sibiricus* accessions, including three early flowering accessions (PI598781, PI655199, LQ10) and three late flowering accessions (PI531665, PI531669, PI595169) were used for transcriptome analysis. Leaf tissues collected at booting stage, heading stage, and flowering stage were used for RNA extraction, cDNA library construction, and Illumina sequencing. These plants were grown in the field plots at the Yuzhong experimental field of Lanzhou University, Gansu, China (latitude 35°34′ N, longitude 103°34’ E, elevation 1720 m). The flag leaf tissues of each accession were collected at three development stage: booting stage, heading stage, and flowering stage.

A total of 18 samples were sent to Breeding Biotechnologies (Xi’an, Shaanxi, China). RNA was extracted with the UNIQ-10 column Trizol total RNA extraction kit (Sangon, Shanghai). cDNA libraries were constructed after the sample is qualified and then all 18 libraries were sequenced using Illumina HiSeq 2500 (data not published) [58]. Raw data were filtered to remove adaptor sequences and low-quality reads to obtain high quality clean data. Unigenes were obtained by de novo transcriptome assembly by Trinity-v2.4.0. Transcripts were annotated by performing BLAST-v2.2.28 against public databases, including Nr (the NCBI non-redundant protein database), Swiss-Prot (Annotated protein sequence database), GO (Gene Ontology), CGO (Clusters of Orthologous Groups), KOG (EuKaryotic Orthologous Groups), KEGG (Kyoto Encyclopedia of Genes and Genomes), and Protein Family (Pfam). We conducted pairwise comparison at three developmental stage between the two genotypes. The differentially expressed genes (DEGs) were filtered with expression levels FDR < 0.01, log2 fold change ≥ 2. The number of unigenes was screened for analysis of SSR using the MISA-v2.1 [59] set to identify mono-, di-, tri-, tetra-, penta-, and hexa-nucleotide motifs. Results of RNA-seq were validated via qRT-PCR experiments.

In this study, candidate genes were homologous genes of the reported flowering genes, which were selected from gene function annotation related to flowering pathways (i.e., vernalization pathway, photoperiodic pathway, gibberellin pathway, autonomic pathway, and age pathway), flowering key genes, and phytohormone responses. Flowering is polygenically controlled and more than 200 predictive genes and other undiscovered flowering genes are involved. Therefore, we expanded the search scope beyond the DEGs and searched the annotation results of the whole unigenes. The candidate gene-based EST-SSR markers were more closely related to flowering regulation than SSR markers. The candidate gene-based EST-SSR primers were designed using Primer 3-v2.3.4 [60] based on the MISA result followed by primer length from 15–30 bp, GC contents from 40–60%, annealing temperature of 60 °C, and no self-complementary sequence. All specific primers were synthesized by the GenScript Company (Nanjing, China).

### 4.3. DNA Extraction, Genotyping and Primer Validation

Fresh leaf tissue of each accession was sampled for DNA extraction using the SDS (sodium dodecyl sulfate) method [61]. DNA quality was determined using the NanoDrop ND1000 spectrophotometer (Thermo Scientific, Waltham, MA, USA). The qualified DNA was diluted to 25 ng/μL and stored at −20 °C. Four DNA samples (Z6, Z9, W2, and W6) with large geographical differences were selected for primers screening.

PCR amplifications were conducted in a volume of 15 μL containing 7.5 μL 2× reaction mix, 5.2 μL ddH_2_O, 0.2 μL golden DNA polymerase (Beijing Tiangen Corporation), 0.05 μL forward primer and reverse primer, and 2 μL of genomic DNA. The PCR conditions were preheat 2 min at 94 °C; 94 °C for 15 s, 10 cycles; annealing temperature 62 °C for 30 s; 72 °C for 30 s; 94 °C for 30 s, 25 cycles; annealing temperature 52 °C for 30 s; 72 °C for 1 min; 72 °C for 7min; and hold at 4 °C. Fragments were separated on 6.0% non-denaturing polyacrylamide gels (PAGE) and visualized using silver staining. In addition, the DL500 DNA marker was used to determine the sizes of the PCR products. Unpurified PCR products were sequenced by Shanghai Sangon Biological Engineering Technology (Shanghai, China) used to the primer validation aligned by MEGA7 software [62].

### 4.4. Allele Scoring and Polymorphism Detection

The polymorphic EST-SSRs closely associated to flowering candidate genes were used for genetic diversity and population structure analysis among 20 *E. sibiricus* accessions including two contrasting panel (early-flowering and late-flowering). The number of alleles of each candidate gene-based EST-SSR locus was calculated based on presence (1) or absence (0). The genetic relationship was analyzed based on a data matrix constructed by EST-SSR markers data using the NTSYSpc-v2.10e software (Exeter Software, Setauket, NY, USA). In addition, a dendrogram was constructed for early-flowering and late-flowering *E. sibiricus* genotypes following the unweighted pair group method analysis (UPGMA) based on Jaccard’s similarity coefficient [63]. Heterozygosity (Ho), gene diversity (He), and the polymorphic information content (PIC) were calculated as previously reported. PIC was calculated for each polymorphic primer using the formula: PIC = 1 − p^2^ − q^2^, where p is the frequency of present band, and q is the frequency of the absent band. Heterozygosity (Ho) is the ratio of heterozygous individuals in the population. At a single locus, the formula is as follows: Ho = $1-\sum_{i=1}^{k}Pluu$. Gene diversity (He) is often referred to as expected heterozygosity. He = $1-\Sigma pi^{2}$, where *pi* is frequency of the *i*th allele [64]. The number of heterozygotes is calculated by the direct count method. The model-based approach implemented was used to subdivide the individuals into different subgroups in the program STRUCTURE-v2.3.2 (Stanford University, CA, USA). The flowering time heatmap of early-flowering and late-flowering *E. sibiricus* germplasms made by Heatmap Illustrator-v1.0 (Huazhong University of Science and Technology, Wuhan, China).

## 5. Conclusions

In this study, 125 candidate gene-based EST-SSRs were developed. Further, 15 EST-SSRs closely associated to 13 candidate genes were used to analyze genetic diversity and population structure among 20 *E. sibiricus* accessions including two contrasting panels (early-flowering and late-flowering). The UPGMA cluster and structure analysis showed that the 20 *E. sibiricus* accessions with similar flowering times tended to group together. These newly developed EST-SSR markers could be useful for molecular markers-assisted selection and germplasm evaluation of the flowering traits in *E. sibiricus*.

## Figures and Tables

**Table 1:** Statistical table of SSR (simple sequence repeat) analysis results.

Searching Item	Number
Total number of sequences examined	40,639
Total size of examined sequences (bp)	78,157,064
Total number of identified SSRs	13,052
Number of SSR containing sequences	10,286
Number of sequences containing more than 1 SSR	2176
Number of SSRs present in compound formation	742
Mono-nucleotide	5471
Di-nucleotide	2642
Tri-nucleotide	4551
Tetra-nucleotide	314
Penta-nucleotide	48
Hexa-nucleotide	26

**Table 2:** He (gene diversity), Ho (heterozygosity), and PIC (polymorphic information content) of 15 polymorphic primers amplified in 20 *E. sibiricus* germplasms.

Primer Code	Flowering Genes	Flowering Pathways/Functions	He	Ho	PIC
Primer 46865	*FPF1*	Flowering	0.6592	0.9231	0.4767
Primer 33680	*FPF1*	Flowering	0.2268	0.1500	0.1275
Primer 43088	*ELF3*	Flowering	0.7966	0.9412	0.4210
Primer 41496	*GID1*	Gibberellin	0.4911	0.5000	0.2367
Primer 40505	*GA2OX6*	Gibberellin	0.6655	0.7000	0.2513
Primer 37852	*CIGR*	Gibberellin	0.1975	0.0588	0.2500
Primer 34975	*XCT*	Circadian Clock	0.5170	0.8000	0.1567
Primer 48198	*GIGANTEA*	Circadian Clock	0.6584	1.0000	0.1400
Primer 34500	*MBD9*	*CONSTANS-Like*	0.8731	1.0000	0.2606
Primer 31670	*NFYC4*	*CONSTANS-Like*	0.3200	0.5946	0.1875
Primer 43287	*COL4*	*CONSTANS-Like*	0.7138	0.9730	0.3325
Primer 36067	*COL13*	*CONSTANS-Like*	0.8117	0.9730	0.3025
Primer 36927	*FLC*	Central Integrator	0.8147	1.0000	0.1500
Primer 34261	*HD3*	Central Integrator	0.7794	1.0000	0.2590
Primer 28366	*HD3a*	Central Integrator	0.6509	1.0000	0.1600
Mean			0.6117	0.7742	0.2475

**Table 3:** The origins, longitude, latitude, and altitude of 20 *E. sibiricus* accessions.

Code	Accession Number	Sample ID	Origins	Longitude/Latitude	Altitude (m)
1	PI598781	Z1	Baikal, Buryat, Russia	-	-
2	PI610876	Z2	-	-	-
3	PI610994	Z3	Siberia, Russia	-	1250
4	PI636676	Z4	Xiahe, Gansu, China	35.19 N, 102.65 E	2720
5	PI655199	Z5	Hongyuan, Sichuan, China	32.08 N, 102.57 E	3280
6	W610305	Z6	Siberia, Russia	-	-
7	W630476	Z7	Kyrgyzstan	-	-
8	HZ03	Z8	Hezuo, Gansu, China	-	-
9	LT05	Z9	Lintan, Gansu, China	-	-
10	LQ02	Z10	Luqu, Gansu, China	-	-
11	PI435088	W1	Ninshan, Xinjiang, China	-	-
12	PI504462	W2	Xining, Qinghai, China	-	-
13	PI531665	W3	Beijing, China	-	-
14	PI531669	W4	Xining, Qinghai, China	-	2400
15	PI595149	W5	Xinjiang, China	44.17 N, 84.57 E	1500
16	PI595156	W6	Xinjiang, China	45.03 N, 81.12 E	1300
17	PI595162	W7	Xinjiang, China	44.12 N, 87.97 E	1680
18	PI595169	W8	Xinjiang, China	43.77 N, 89.45 E	1300
19	PI595174	W9	Xinjiang, China	43.68 N, 89.30 E	1870
20	PI655092	W10	Mongolia	49.80 N, 94.96 E	1370

## Supplementary Materials

The following are available online at https://www.mdpi.com/2223-7747/9/10/1371/s1. Table S1: Summary of the sequence data analysis. Table S2: Statistics of Unigene Library of *E. sibiricus.* Table S3: BLAST analysis of the non-redundant unigenes against public databases. Table S4: The unigene sequence of 155 candidate genes. Table S5: Information of flowering candidate genes and gene-based EST-SSRs primers: reverse and forward sequence, Tm, molecular weight of expected band. Table S6: The coefficient of genetic similarity of 20 *E. sibiricus* accessions.
